# Evaluating a Dutch cardiology primary care plus intervention on the Triple Aim outcomes: study design of a practice-based quantitative and qualitative research

**DOI:** 10.1186/s12913-017-2580-x

**Published:** 2017-09-06

**Authors:** Tessa C.C. Quanjel, Marieke D. Spreeuwenberg, Jeroen N. Struijs, Caroline A. Baan, Dirk Ruwaard

**Affiliations:** 1 Department of Health Services Research, Care and Public Health Research Institute, Faculty of Health, Medicine and Life Sciences, Maastricht University, P.O. Box 616, 6200 MD Maastricht, the Netherlands; 2 Research Centre for Technology in Care, Zuyd University of Applied Sciences, Heerlen, the Netherlands; 3 Department for Quality of Care and Health Economics, Centre for Nutrition, Prevention and Health Services, National Institute for Public Health and the Environment, P.O. Box 1, 3720 BA Bilthoven, The Netherlands; 4 Scientific Centre for Transformation in Care and Welfare (Tranzo), University of Tilburg, Tilburg, The Netherlands

**Keywords:** Primary care, Primary care plus, Hospital care, Triple Aim, Substitution, Referral, Cardiology

## Abstract

**Background:**

In an attempt to deal with the pressures on the health-care system and to guarantee sustainability, changes are needed. This study focuses on a cardiology primary care plus intervention. Primary care plus (PC+) is a new health-care delivery model focused on substitution of specialist care in the hospital setting with specialist care in the primary care setting. The intervention consists of a cardiology PC+ centre in which cardiologists, supported by other health-care professionals, provide consultations in a primary care setting. The PC+ centre aims to improve the health of the population and quality of care as experienced by patients, and reduce the number of referrals to hospital-based outpatient specialist care in order to reduce health-care costs. These aims reflect the Triple Aim principle. Hence, the objectives of the study are to evaluate the cardiology PC+ centre in terms of the Triple Aim outcomes and to evaluate the process of the introduction of PC+.

**Methods/Design:**

The study is a practice-based, quantitative study with a longitudinal observational design, and an additional qualitative study to supplement, interpret and improve the quantitative study. The study population of the quantitative part will consist of adult patients (≥18 years) with non-acute and low-complexity cardiology-related health complaints, who will be referred to the cardiology PC+ centre (intervention group) or hospital-based outpatient cardiology care (control group). All eligible patients will be asked to complete questionnaires at three different time points consisting of questions about their demographics, health status and experience of care. Additionally, quantitative data will be collected about health-care utilization and related health-care costs at the PC+ centre and the hospital. The qualitative part, consisting of semi-structured interviews, focus groups, and observations, is designed to evaluate the process as well as to amplify, clarify and explain quantitative results.

**Conclusions:**

This study will evaluate a cardiology PC+ centre using quantitative and supplementary qualitative methods. The findings of both sub-studies will fill a gap in knowledge about the effects of PC+ and in particular whether PC+ is able to pursue the Triple Aim outcomes.

**Trial registration:**

NTR6629 (Data registered: 25-08-2017) (registered retrospectively).

**Electronic supplementary material:**

The online version of this article (10.1186/s12913-017-2580-x) contains supplementary material, which is available to authorized users.

## Background

In an attempt to deal with the pressures on the health-care system and to guarantee sustainability, changes are needed [1, 2]. The aging population, along with an increase in chronically ill patients and medical and technological developments are expected to cause an even further increase in the health-care expenditures in most Western countries [3–5]. To guarantee sustainability, the health-care system should focus on high-value health care. This implies that initiatives should focus on simultaneously pursuing the three aims: improving the health of the population, improving the quality of care (as experienced by patients) and reducing the increase of health-care costs. This is known as the ‘Triple Aim’ principle of Berwick and colleagues [6, 7].

During the past few years, the Dutch Ministry of Health has determined strategies to limit the growth of health-care costs and to increase quality of care [2, 8–12]. A concrete action in line with these strategies includes that the Ministry of Health appointed nine regional initiatives as ‘pioneer sites’. These pioneer sites are able to experiment with (new) interventions which are focused on the regional population. They all aim to restructure health services based on the concept of population management (PM). Although an unambiguous and widely accepted definition of PM is lacking, PM initiatives focus on addressing health needs at all points along the continuum of health and well-being for a specified population by integrating services across health care, prevention, social care and welfare [13]. Additionally, these pioneer sites follow the abovementioned ‘Triple Aim’ principle [6, 7]. A considerable number of the interventions implemented in the pioneer site regions are focussing on substitution of care. Substitution of care can be defined as the continual regrouping of resources across and within care settings to exploit the best and least costly solution in the face of changing needs and demands [14]. All pioneer sites will be monitored over the coming years.

This paper concerns a practice-based study focused on a cardiology primary care plus centre which is implemented in one of the appointed pioneer sites. The pioneer site, named ‘My Care’, as well as the intervention, is located in the southern part of the Netherlands [19]. In the Netherlands, all residents are mandated to purchase insurance policies, which cover an essential-benefits package. The majority of the Dutch hospitals are not-for-profit organizations and virtually all specialists are hospital-based. GPs act as gatekeepers of the health-care system and promote consistency and coordination of individual care [15–17]. All residents are registered with a GP and have unlimited access to the GP. Hospital and specialist care is only accessible through GP referral, with the exception of emergency care. In addition, when a patient with a particular complaint is referred by a GP to hospital-based specialist care, the specialist can refer the patient, for that particular complaint, to another specialist [16, 17]. It is also important to mention the influence of the different payment systems for Dutch health-care professionals. On the one hand, the GP payment system is a blended model that combines a capitated fee per enrollee with a relatively small fee for service payment per visit [15]. This blended GP payment system does not incentivize GPs to maximize the volume of care and does not encourage GPs to refer patients to specialist care. On the other hand, the payment system of the hospitals is based on a Diagnostic Related Groups (DRGs)-type system. Specialist fees are integrated into DRGs. Hospitals, and self-employed specialists, receive a payment for each DRG. This payment system incentivizes specialists to increase the volume of hospital care by increasing the number of cases, i.e. DRGs [15, 18].

The practice-based study focuses on a cardiology Primary Care Plus (PC+) centre. In this pioneer site PC+ implies substitution of specialist care in the hospital setting with specialist care in the primary care setting and it is designed for patients with non-acute and low-complexity related health complaints. Since virtually all specialists are hospital-based in the Netherlands, PC+ is a new form of health care. In the cardiology PC+ centre cardiologists provide consultations in the primary care setting. During the PC+ consultation the patients receive a comprehensive screening and afterwards the cardiologist sends his recommendations for further treatment (if needed) to the GP. For example, the specialist informs the GP about whether a referral to hospital care is needed. Based on the recommendation, the GP discusses the options for further treatment with the patient. Moreover, within PC+ the GP remains clinically in charge of the patient. PC+ strengthens the gatekeeping and coordinating role of the GP by intensifying the collaboration and communication between the specialists and the GPs. The PC+ intervention aims to improve the health of the population and patients’ experience of care and to reduce the number of referrals to hospital care (as well as hospital-based outpatient specialist care) in order to reduce health-care costs growth. In this study, the PC+ intervention is compared to care-as-usual, both are described in detail in the methods section. This paper describes the design of the evaluation of the cardiology PC+ intervention, combining qualitative and quantitative methods.

### Objectives and underlying premises of PC+

This overall study aims to evaluate the effects of cardiology PC+ centre on the Triple Aim outcomes. The underlying premises of the researchers on the effects of the intervention are that the cardiology PC+ centre will result in an (at least) equivalent health of the population, improved quality of care as experienced by patients and a reduced number of referrals to hospital-based outpatient cardiology care, and hence reduced health-care costs.

The underlying premises are grounded on several hypotheses. Firstly, it is supposed that the health of the study population (patients with non-acute and low-complexity cardiology complaints) will be at least the same, since the patients receive the same diagnostic tests and the care is provided by health-care professionals with the same level of expertise. Thus, the cardiology PC+ centre and hospital-based outpatient cardiology care will lead to the same health outcomes. After the introduction of PC+, the patients who need more specialized hospital care are likely to be referred by their GP to the hospital and the patients who do not need hospital care will remain in primary care.

Secondly, the aim of the introduction of PC+ is to improve patients’ experience of care. In this study the component patients’ experience of care is based upon the six dimensions of health-care performance of the Institute of Medicine (IOM), which partially overlaps with the last premise about health-care costs [19]. It is supposed that PC+ will improve: 1) the effectiveness of care since PC+ will result in fewer unnecessary referrals to hospital-based outpatient cardiology care, thus less overuse of care, 2) the timeliness of care since PC+ will decrease the waiting time, 3) the patient-centredness of care since an underlying rationale of PC+ is being respectful and responsive to individual patient preferences, needs and values, and 4) the efficiency of care since PC+ will result in less waste of expensive hospital-based outpatient cardiology care. Efficiency of care is related to health-care costs, therefore it will be included in this dimension. In addition, it is supposed that PC+ has no effect on the level of 5) safety of care and 6) equity of care and thus it is supposed that these will remain at the same level.

Finally, it is supposed that the introduction of PC+ will lead to reduced health-care costs by: 1) fewer referrals to hospital-based outpatient care because of the introduction of PC+, 2) lower costs in PC+ than in hospital-based outpatient care, due to lower overhead costs (i.e. the same cardiology screening will costs less in PC+), 3) less use of additional hospital-based services because it is supposed that a significant number of the patients referred to PC+ will not be referred to hospital care afterwards, but will remain in the primary care setting, and 4) GPs will gain more knowledge because of the enhanced collaboration and communication with the cardiologists. Hence, in the long term, it is possible that they will refer fewer patients, to either PC+ or hospital-outpatient care, for a cardiology screening and instead treat the patients themselves.

## Methods

This clinical observational study will adhere to the SPIRIT guidelines. The SPIRIT checklist will be used and it is added as supplementary [see Additional file 1]. The following paragraphs will describe the intervention versus care-as-usual, the study design and the methods of this research.

### The pioneer site

The ‘My Care’ region is a geographically demarcated region, located in the most southern part of the Netherlands. It consists of 11 municipalities, 277,000 residents, approximately 135 GPs and one hospital. The region is characterized by a relatively unhealthy population with a low socio-economic status (SES) as compared to the overall population of the Netherlands [20]. Moreover, it has the highest percentage of patients with a chronic disease in the Netherlands, namely 68.5% [20]. This results in a relatively high demand for health care and thereby high health-care expenditures, especially for chronic care, diagnostics and medication [21, 22].

The pioneer site ‘My Care’ is a partnership between the care group called ‘General Practitioners Eastern South-Limburg’, which is a legal entity of all GPs in the region (in Dutch: Huisartsen Oostelijk Zuid-Limburg) [23], the regional hospital Zuyderland Medical Centre, the patient representative foundation called ‘House for Care’ (in Dutch: Huis voor de Zorg) and the dominant health insurance company in the region (named: CZ*)*.

### The intervention: the primary care plus centre

**Fig. 1:**
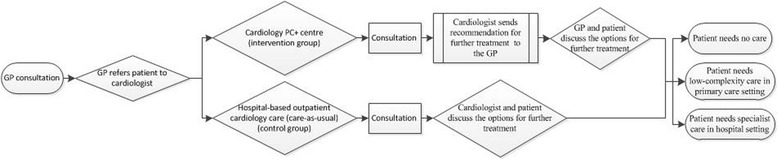
Flowchart: showing the flow of the intervention group and control group

The PC+ intervention is a cardiology PC+ centre in which cardiologists, supported by other health-care professionals, provide specialist consultations in a primary care setting. Hospital diagnostic tools are available including an ultrasound device, an ergometer and an electrocardiogram (ECG). All GPs in the region participate in the PC+ intervention and are able to refer non-acute and low-complexity patients with cardiology-related complaints to the PC+ centre. Patients who are already diagnosed with cardiology-related health problems by a cardiologist are not appropriate for PC+ and (if needed) they will be treated by the cardiologist in the hospital-care setting. The consultation at the PC+ centre consists of the following diagnostic tests: a blood test, an ECG, an echo and an exercise test. The diagnostic tests are carried out by multiple health-care professionals, such as nurses, laboratory technicians and physicians, all specialized in cardiology. After the tests the patient meets the cardiologist, who explains the results of the diagnostic tests. The cardiologist sends a comprehensive description of the results of the tests, the diagnosis and his recommendation regarding further treatment (if needed) to the GP. The GP discusses the cardiologist’s recommendation with the patient and based on the principles of shared decision-making the GP and the patient discuss the options for further treatment [24]. Moreover, the GP remains clinically in charge of the patient. The further treatment will depend on the results of the tests and the recommendation of the cardiologist; the three overall options are: 1) the patient needs no care (i.e. the patient has no health problems that need further attention), 2) the patient will remain in the primary care setting (the patient needs low-complexity care, e.g. medication) or, 3) the patient will be referred to secondary care (the patient needs specialist care). The flow of patients is illustrated in Fig. 1.

### Care-as-usual: hospital-based outpatient cardiology care

Hospital-based outpatient cardiology care is considered care-as-usual. All GPs are allowed to refer non-acute and low-complexity patients with cardiology-related complaints to the hospital-based outpatient cardiology care. These patients receive the same care as within the PC+ centre, i.e. the same diagnostic tests carried out by health-care professionals with the same level of expertise. After the tests the patient meets the cardiologist, the cardiologist explains the results and they discuss further treatment (if needed). This underlines a significant difference with the intervention: in the PC+ centre the cardiologist provides only a recommendation on further treatment and the GP discusses the options for further treatment (instead of the cardiologist), i.e. within PC+ the GP remains in charge. The flow of patients is illustrated in Fig. 1.

### Study design

The study is a practice-based, quantitative study with a longitudinal observational design, and an additional qualitative study to supplement, interpret and improve the quantitative study. The research study is, at its core, a quantitative study with qualitative data added to provide an added value and deeper, wider and fuller answers to the research questions. These interrelated and reinforcing sub-studies are split up as follows:

1. A quantitative longitudinal observational study aimed at measuring the effects of PC+.
2. A qualitative study to evaluate the process of the introduction of PC+ as well as to amplify, clarify and explain quantitative results.

### Sub-study 1: the quantitative study

#### Aim

The aim of the quantitative part is to measure the effects of the PC+ intervention on the population’s health, patients’ experience of care and health-care costs. To measure the effects the researchers will use the following sources: patient questionnaires, data from the cardiology PC+ centre, data from the regional hospital and health insurance claims data. The data will be collected at four different time points, namely at baseline, before the patient has the first consultation with the cardiologist (T0), a week after the first consultation (T1), 3 months after the first consultation (T2) and 6 months after the first consultation (T3). The outcome parameters, including the dimensions, outcomes, concepts, methods and time frame, are summarized in Table 1.

**Table 1:** Overview of the outcome measurements

Dimension	Outcome measure	Concept	Method	Data collection time			
				T0	T1	T2	T3
Population’s health	Health status, health-related quality of life	EQ-5D-5 L	Questionnaires	X	X	X	
		EQ-VAS		X	X	X	
		SF-12		X		X	
Experience of care	Effectiveness	EQ-5D-5 L	Questionnaires	X	X	X	
		Number of referrals	Data^a^	X		X	X
	Timeliness	Time between referral of the GP and appointment at the PC+ centre/hospital	Questionnaires	X	X		
	Patient centred	Questions about the experiences of patients with health care	Questionnaires		X		
	Safety	% Hospital admissions	Data^a^				X
		% Emergency care visits					X
	Equity	Subgroup analysis, e.g. educational level and age	Questionnaires	X		X	
Health-care costs	Health-care costs: primary care, PC+, secondary care and diagnostics	Health insurance claims data	Data^a^				X
	Efficiency	Number of consultations	Data^a^				X
		% Hospital admissions					X
		% Patients referred to hospital after PC+					X

T0 – at baseline: before the consultation

T1 – within a week after the consultation

T2 – 3 months after the consultation (follow-up)

T3 – 6 months after the consultation (follow-up)

^a^Data of the PC+ centre, the hospital and Vektis

### Study population

The study population consists of adult patients (≥18 years) with non-acute and low-complexity cardiology-related health complaints, registered with a GP in the region of the pioneer site. Based on the principles of shared decision-making the GP and the patient will discuss the options for referral which will be either the PC+ centre (intervention group) or the hospital-based outpatient cardiology care (control group). The decision will be based on expertise and experience of the GP, the severity of the complaints of the patient and the preferences of the patient. Less sever, non-acute and low-complex patients should be referred to PC+. At the moment, no specific criteria are included for the referral to PC+. The GP is in charge of referring a patient to the PC+ centre or the hospital-based outpatient cardiology care, i.e. the researchers do not have any influence on the referrals.

Excluded from participation are patients who are already diagnosed with cardiology-related health problems by a cardiologist and patients who have received balloon angioplasty or bypass surgery in the past 18 months. Furthermore, patients with acute health problems who require immediate hospital care and/or patients arriving at the emergency department of the hospital are excluded from participation.

### Recruitment of patients

**Fig. 2:**
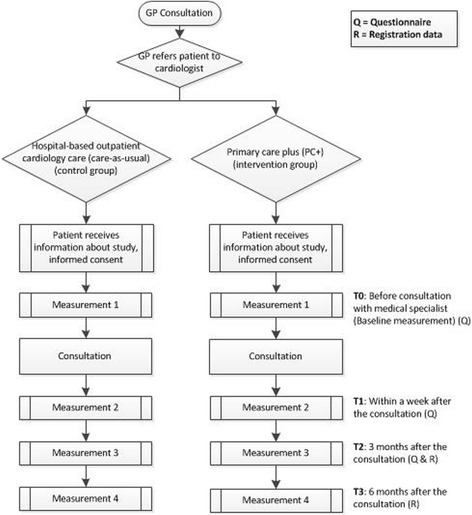
Flowchart: flow of the participants and measurements

Regarding the allocation of patients, this is a practice-based study and therefore the allocation of patients is not random but based on the decision of the GPs. The patient enrolment of both the intervention group and the control group starts when a GP refers a patient to PC+ or hospital-based outpatient cardiology care. Furthermore, it will be statistically tested whether the groups differ in patients’ characteristics at baseline. In case the there is a difference in patients’ characteristics at baseline, the propensity score method (PSM) is used to statistical control for the differences [25]. In addition, the GP will inform all patients about the study. Before the consultation at the PC+ centre or the hospital the patients will receive an information letter with an informed consent form. In this letter they are asked if they would like to participate in the study. All eligible patients have to give informed consent. The flow of participants and the time of measurements, i.e. data collection, are summarized in Fig. 2.

### Data collection

#### Patient questionnaire

All participating patients will be asked to complete questionnaires at three different time points, namely at baseline (e.g. before the consultation with the cardiologist (T0)), within a week after the consultation (T1) and at three-month follow-up after the consultation (T2). The baseline questionnaire (T0) consists of questions about the demographics and health of the patient. Questionnaire 2 (T1) consists of questions about the health of the patient and about the patient’s experience of care. Questionnaire 3 (T2) consists only of questions about the health of the patient. All patients who signed the informed consent, but of whom the researchers did not receive the questionnaire yet, will be kindly reminded to complete the questionnaire. Patients will receive up to two reminders, one by phone and one by a regular mail. Moreover, all outcome parameters are explained in one of the following sections.

#### Data from the cardiology PC+ centre

Data registered by the PC+ centre include the following information about each patient and their consultation: gender, date of birth, name of referring GP, referral date, reason(s) for referral (including signs and symptoms of the patient), consultation date, name of the treating cardiologist, content of the consultation, recommendation regarding further diagnostics, treatment or referral, added value of the consultation, and whether the patient’s health needs were properly addressed. The starting point is the date of referral to the PC+ centre with a follow-up period of 6 months. All data will be collected by the health-care professionals of the PC+ centre, recorded in one document and exchanged with the researchers.

#### Data of the regional hospital

Hospital data include two types of patients: 1) control group patients and 2) intervention group patients (patients who have had a consultation at the PC+ centre and are referred to the hospital afterwards). The collected data are similar to the data of the PC+ centre. The starting point of the data collection is the date of referral to the PC+ centre or the hospital-based outpatient cardiology care with a follow-up period of up to 6 months. In addition to the above-mentioned collected patient information, the following hospital data will be provided: the number of consultations, the number of hospital admissions and the number of emergency care visits of a patient over a period of 6 months. The data are extracted from the registration system of the hospital.

#### Health insurance claims data

This study will only focus on the health-care costs of patients, which will be measured using the health insurance claims data of the participants, which are available at Vektis. Vektis is a national information centre for all the health insurance companies in the Netherlands. The centre collects all data on the health-care utilization and health-care costs of all Dutch residents [26]. Health insurance claims data will be used to examine whether the health-care costs of an intervention patient (PC+) is different to the health-care costs of a control patient. To compare the health-care costs of an intervention patient with that of a control patient, the health insurance claims of each patient participating in the intervention or control group will be used, with a focus on GP care, PC+ and hospital care. Moreover, it has been decided to take into account the health-care costs per participant over a period of 6 months. The starting point is the date of referral to the PC+ centre (intervention group) or the hospital-based outpatient cardiology care (control group).

### Outcome parameters

The outcome parameters are related to the Triple Aim principle and are focused on (1) population health, (2) experience of care and (3) health-care costs [6]. All outcome parameters of the quantitative study, including the dimension, outcome, concept, method and time of data collection, are summarized in Table 1. The outcome parameters, related measures and selected questionnaires are based upon suggested measures by the Institute of Healthcare Improvement to operationally define and measure the Triple Aim [27].

#### Population health

Population health will be measured with three generic validated questionnaires, namely the EQ-5D-5 L, the EQ-VAS and the Short Form Health Survey (SF-12).

The EQ-5D-5 L incorporates descriptions and valuations of health status and reflects how patients value their own health state. The questionnaire comprises the dimensions mobility, self-care, usual activities, pain/discomfort and anxiety/depression [28, 29]. The scores range from −0.33 to 1 (worst imaginable to best imaginable health state), using the Dutch utility tariff [30]. Instead of the EQ-5D-3 L 3-level version, we have chosen to use the EQ-5D-5 L 5-level, because this appears to be a valid extension of the 3-level. Several studies indicate that the measurement properties of the EQ-5D-5 L are superior to the EQ-5D-3 L in terms of feasibility, reliability, ceiling effects, discriminatory power and convergent validity [31–33].

The EQ-VAS (visual analogue scale) is added; this is a reflection of how patients value their own health state (range from 0 to 100).

The SF-12 is a subset of the larger SF-36 [34]. The questionnaire comprises eight health aspects, namely physical functioning, role limitations because of physical health problems, bodily pain, general health, vitality (energy/fatigue), social functioning, role limitations because of emotional problems, and mental health (psychological distress and psychological well-being) [34–37]. The reliability of the SF-12 is high (> 0.80) [34–37]. In addition, three items from the SF-36 are added to calculate the Mental Health Inventory score, comprising the dimensions feeling nervous, feeling downhearted and blue, and feeling happy [34].

#### Experience of care

The experience of care will be measured using the perspective of the patient, i.e. the patient’s interaction and experience with the health-care system. The outcome parameters are based upon the six dimensions of health-care performance of the IOM: safety, effectiveness, equity, timeliness, patient-centredness and efficiency [19]. As mentioned above, efficiency will be included in the dimension health-care costs.

Effectiveness of care will be measured with the EQ-5D-5 L questionnaire and the percentage of referrals to hospital-based outpatient cardiology care. On the one hand, effectiveness will be measured using the EQ-5D-5 L scores, where it is analysed whether the health of the population is improved after a follow-up of 3 months. On the other hand, effectiveness will be measured using the number of referrals to hospital-based outpatient cardiology care, where it is analysed whether this number is decreased in the period from 2013 to 2017.

Timeliness of care will be examined by measuring the waiting time (in days) between the day the patient is referred by his GP and the day of the first consultation with the cardiologist.

Patient-centred care will be measured using items based on the Consumer Quality (CQ) index. The CQ index is a standardized method for measuring experiences of patients with health care [38]. The questionnaire (T1) includes 30 questions about the experiences of the patients with health care, with specific regard to their experiences with the health-care professionals (e.g. *‘Did you feel welcome and at ease?’* and *‘Did the health-care professionals listen attentively?’*).

Safety of care will be measured using data that will be made available by the hospital concerning: 1) hospital admissions; 2) emergency care visits.

Equity of care will be examined by performing subgroup analysis on all outcome measures regarding educational level (high versus low) and age (lower than median and higher than median).

#### Health-care costs

Health-care costs will be measured using health insurance claims data. This study will not cover all related health-care costs but will only focus on health-care costs (including patients’ compulsory deductible). Out-of-pocket payments and indirect costs made from a societal perspective (e.g. labour market, informal care) will not be included. The health-care costs per participant is measured over a period of 6 months after the consultation at the PC+ centre or the hospital-based outpatient cardiology care. This concerns data regarding health-care costs on primary care, PC+, hospital care and diagnostics.

Efficiency of care means, according to the IOM, avoiding waste, particularly waste of equipment, supplies, ideas and energy [19]. With regard to this research, it will be measured whether the number of consultations of patients, hospital admissions and referrals will decrease after the implementation of PC+, which is related to efficient use of equipment and health-care professionals and time of patients. Efficiency of care will be measured using data from the cardiology PC+ centre, the hospital and Vektis. The data will consist of the following items: 1) the average number of consultations at the hospital of a patient over a period of 6 months; 2) the average number of hospital admissions of a patient over a period of 6 months; and 3) the number and percentage of patients that are referred to hospital care after PC+.

### Sample size and power calculation

The sample size and power calculation is based on the referral data of the hospital in 2013 extracted from its registration system. The data of 2013 show that 12% (1755 referrals to hospital-based outpatient cardiology care) of all referrals (14,978 referrals) to the cardiology department of the hospital are eligible for PC+. These eligible patients are referred to hospital-based outpatient cardiology care, which provides, among other health care, the same health care as the PC+ centre. A decrease of 50% in the number of referrals to hospital-based outpatient cardiology care is seen as a relevant difference. So, the number of referrals eligible for PC+ will decrease from 1755 (12% of the total referrals to cardiology) referrals to 878 (6% of the total referrals to cardiology). While assuming a power of 80% and a significance level of 0.05, 358 patients per group (intervention as well as control) are required. Taking into account a dropout of 20%, a sample size of 429 patients per group is required, so this study needs to include 858 patients in total.

To calculate the sample size the following formula was used to compare proportions:

$$ N=\frac{{\left({Z}_{\alpha }/2+{Z}_{\beta}\right)}^2\ast p\kern0.2em \left(1-p\right)\left(r+1\right)}{{\left({p}_{0-}\kern0.2em {p}_1\right)}^2\ast r} $$

With the following meaning of the abbreviations:

**Table:** 

Zα/2 = critical value of the normal distribution at α/2; α = 0.05	1.96
Zβ = critical value of the normal distribution at β; β is 0.2	0.84
p0 = expected proportion of referrals eligible for PC+ at baseline	0.12 (12%)
p1 = expected proportion of referrals eligible for PC+ 1 year after the intervention is implemented	0.06 (6%)
p = (p1+ p0)/2	0.090
r = ratio of patients per group	1

### Planned statistical analysis

In general, the continuous outcome measures will be expressed in means, medians and standard deviations and the categorical variables in numbers and percentages.

#### Descriptive statistics

Demographic data (e.g. gender, age, level of education, nationality and co-morbidities) will be described for the total group and for the intervention and control group, separately. The two groups will be tested on differences between characteristics, using the t-test for continuous variables and the chi-square test for categorical variables. If variables differ between the two groups, with a *p*-value ≤0.10 they are considered to be potential confounders in further analysis.

#### Data analysis

To investigate the effect of PC+ on population health, patients’ experience of care and health-care costs an intention to treat analysis will be conducted. The between-group comparisons will be analysed with multilevel analysis to account for the dependency of observations in time. We will apply a two-level linear mixed model (time, participants) and the level of statistical significance will be set at 0.05 (two-tailed). Separate models (random intercept) will be set up for each outcome measure. The independent variables in each model are a dummy variable indicating the group (control group = reference category), three dummy variables for time (T0 = reference category) and the interaction variable between group and time.

To correct for the possible confounding influence of baseline characteristics of the patients (e.g. age, gender, level of education, native country and health-care costs) and the GP characteristics (e.g. age, gender and practice) a propensity score will be added into the multilevel model. The propensity score will be estimated using a logistic regression model with treatment group (control or intervention) as dependent variables and baseline variables (patient and GP characteristics) included as independent variables. Only baseline variables that are related to the outcome variable (with a *p*-value smaller than 0.10) are included in the propensity score [25].

If normality assumptions are violated, outcome variables will be log-transformed and if necessary non-parametric tests will be used.

SPSS version 22 is used to analyse the data.

### Procedure for accounting for missing data

The missing values on items in the questionnaires will be handled according to the scoring algorithms of the questionnaires. Since it has been shown that multilevel analysis is a flexible method for handling missing data for repeated-measures designs, the missing variables in the follow-up data will not be imputed [39]. The other missing values, for non-repeated measures, will be handled according to multiple imputation, which means that missing values will be predicted using existing values from other variables.

### Stopping rules

There are no formal statistical stopping rules. Patients can withdraw from the study at any time.

### Sub-study 2: the qualitative study

#### Aim

The qualitative study will consist of semi-structured interviews, focus groups and observations. Besides evaluating the process of the introduction of PC+ (e.g. identifying the barriers and facilitators), the aim of the qualitative study is to clarify and explain quantitative results. Therefore, the qualitative study will be based on an adaptive approach; the ultimate design will depend on developments during the research and results of the quantitative study.

### Study population

The study population of the qualitative study will focus on the different stakeholders of the PC+ centre, namely the manager of the PC+ centre, the head of the cardiology department of the hospital, GPs, cardiologists, members of the Steering Committee of the pioneer site ‘My Care’ and patients. In addition, interviews and focus groups will be held with patients. The patients will be recruited purposively in order to generate a divergent sample of the patient population. The patient population of the qualitative study will consist of: patients who had a consultation at the PC+ centre, patients who had a consultation in regular hospital-based outpatient care and patients who had a consultation at the PC+ centre and are referred to hospital care afterwards.

### Data collection

The semi-structured interviews and focus groups will include the following topics: development, content, goals and targets of the PC+ intervention, facilitators and barriers of the introduction of PC+, expected results, (personal) experiences and, responses and opinions regarding certain (quantitative) results. The interviews and focus groups will be held by one interviewer and one observer, and will be audio-recorded. In addition, the researchers will observe the monthly meetings of the Steering Committee to collect information about the barriers and facilitators of the introduction of PC+. In general, the number of interviews, focus groups and observations will be based on information saturation. Moreover, the qualitative study will be based on an adaptive approach because the data collection of the qualitative study is partly based on the quantitative results.

### Data analysis

The recorded interviews will be transcribed verbatim by the researchers using the transcription instructions of Poland [40]. The interviews, focus groups and observations will be analysed using conventional content analysis methods and if it is assumed to be appropriate, open colour coding and axial coding methods will be used [40, 41]. The program NVivo10 will be used as a supportive tool. All qualitative data will be analysed independently by at least two researchers. Afterwards, the researchers will discuss the results. Moreover, the results will also be validated by performing member checks, which means that the findings will be discussed with the particular respondents.

## Discussion

Primary care plus (PC+) is a new concept in the Dutch healthcare system but its support is largely based on conceptual grounds and evidence about the process of implementation and the effects is scarce. Therefore, we will perform a quantitative longitudinal observational study with an additional reinforcing qualitative study. The quantitative part focuses on providing insights into the effects of the PC+ intervention, in terms of the population health, quality of care as experienced by patients and health-care costs. Additionally, the qualitative part focuses on evaluating the process of the introduction of PC+ and clarifying and explaining the quantitative results. The use of both research methods will result in a broader view for the researchers and it will add value to the results of this study. For example, the qualitative data will result in extensive information about the implementation process of PC+, which will support the researchers in interpreting the quantitative results.

This study is a practice-based research. Nowadays, practice-based research is seen as a viable alternative for randomized controlled trial design [41, 42]. Practice-based research has a considerable value because it ensures connections between science, policy and practice. A major advantage of practice-based research is that it results in evidence-based practice, which means that research findings can be (directly) translated into policy and practice. Moreover, the external validity of practice-based research is commonly higher than that of randomized control trials, since the results are more generalizable [41, 42].

The study design has some limitations of which the lack of non-random selection of patients is the most prominent. Patients will be selected by GPs. The fact that the selection of the groups is determined by the (unobservable) choice of the GP may cause selection bias. However, selection bias will be limited as the groups will be equated by the strict prescribed in,- and exclusion of, the PC+ centre and by using the propensity score method (PSM) [25]. The PSM reduces the entire collection of observed baseline variables to a single score. The estimated propensity score is defined as the conditional probability of assignment to a particular group, given a set of observed baseline characteristics [25, 43]. PSM offers statistical control over observed baseline differences between patients in a non-randomized study [25].

The pioneer site ‘My Care’ is engaged in expanding the PC+ centre with more medical specialities. At present the centre only offers cardiology care, but in the near future they will also focus on the following four specialities: dermatology, minor surgical operation, internal medicine and ear, nose and throat care. Consequently, this research will also expand, with similar studies with regard to the added specialities. This will also improve this study, since the expanding makes it possible to examine whether the effectiveness of PC+ is related to the type of speciality. For example, it is possible that PC+ is effective within the speciality of dermatology but not within the speciality of surgery. Additionally, the results of this study will lead to recommendations for the pioneer site. The pioneer site can use the recommendations in the design of the expansion of the PC+ centre with other specialities.

As described in the introduction section, the Dutch Ministry of Health appointed nine regions as pioneer sites. All pioneer sites strive to accomplish high-quality improvements in health care by pursuing the Triple Aim principle. This intervention is part of one of the pioneer sites, named ‘My Care’. In the future, it would be interesting to exchange information about the results of PC+ interventions across pioneer sites and also of other PC+ interventions in the Netherlands. For example, it would be interesting to perform a case study to investigate the successes and failures of the PC+ interventions within the Netherlands. These findings can be extremely useful; as a matter of fact, this would make it possible to mutually learn from each other. Moreover, most Western countries attempt to deal with similar pressures on the health-care system and therewith the increase of the health-care costs [1, 4, 5]. Additionally, health-care initiatives focused on the Triple Aim principle are adopted globally across all health-care systems, for different contexts and settings [44]. Hence, our findings might also be of importance for other countries or regions. If the PC+ intervention demonstrates progress in the Triple Aim, this could mean that it is also worth other countries and regions considering the implementation of PC+ interventions.

## Conclusion

In conclusion, this practice-based study, using both quantitative and qualitative methods, will evaluate the relatively newly introduced concept of PC+. This study will focus on a cardiology PC+ centre and the findings will fill a gap in knowledge about the effects of PC+ and in particular whether PC+ is able to pursue the Triple Aim outcomes.

## Additional file

Additional file 1: SPIRIT Checklist. (PDF 121 kb)

## Abbreviations

DRG: Diagnostic Related Group
ECG: Electrocardiogram
GP: General practitioner
IOM: Institute of Medicine
PC+: Primary care plus
PSM: Propensity score method
SES: Socio-economic status
